# The Endometrial Immune Profiling May Positively Affect the Management of Recurrent Pregnancy Loss

**DOI:** 10.3389/fimmu.2021.656701

**Published:** 2021-03-24

**Authors:** Meryam Cheloufi, Alaa Kazhalawi, Anne Pinton, Mona Rahmati, Lucie Chevrier, Laura Prat-ellenberg, Anne-Sophie Michel, Geraldine Dray, Arsène Mekinian, Gilles Kayem, Nathalie Lédée

**Affiliations:** ^1^Department of Obstetrics and Gynecology, Trousseau Hospital, APHP, Sorbonne Université, FHU PREMA, Paris, France; ^2^MatriceLAB Innove SARL, Pépinière Paris Santé Cochin, Paris, France; ^3^London Women’s Clinic, London, United Kingdom; ^4^Centre d’Assistance Médical á la Procréation Bluets-Drouot, Hôpital Les Bluets, Paris, France; ^5^Hôpital Saint-Antoine Groupe Hospitalier AP-HP, Sorbonne Université (Paris), Paris, France

**Keywords:** recurrent pregnancy loss (RPL), uterine immune profile, embryo implantation, endometrium, Assisted Reproductive Technology (ART)

## Abstract

**Introduction:**

The endometrial immune profiling is an innovative approach based on the analysis of the local immune reaction occurring in the endometrium at the time of the embryo implantation. By documenting the local immune activation during the period of uterine receptivity, we aim to detect and correct potential imbalances before and at the very beginning of placentation. The main objective of the study was to analyze in women with a history of repeated pregnancy loss (RPL) the association of personalized strategies based on immune dysregulations with live birth rates. The secondary objective was to highlight the main prognostic factors for live births.

**Methods:**

This is an observational retrospective analysis of 104 patients with RPL, included between January 2012 and December 2019. Inclusion criteria included a spontaneous fertility with at least three miscarriages, an assessment including a three-dimension ultrasound scan, an endometrial biopsy for uterine immune profiling and a follow-up over at least 6 months with personalized care if indicated after the complete assessment. We defined as a success if the patients had a live birth after the suggested plan, as a failure if the patient either did not get pregnant or experienced a new miscarriage after the targeted therapies.

**Results:**

Uterine immune profiling was the only exploration to be significantly associated with a higher live birth rate (LBR) if a dysregulation was identified and treated accordingly (55% vs 45%, p=0.01). On the contrary, an absence of local dysregulation (resulting in an apparently balanced immune environment) was associated with a higher risk of a new miscarriage, suggesting that the cause inducing RPL still needed to be identified. Independently of age and AMH level, dysregulated immune profile is significatively associated with 3 times higher LBR than a non-deregulated profile (OR=3.4 CI 95%1.27-9.84) or five times in case of an overactive profile treated by immunotherapy (OR=5 CI 95% 1.65-16.5). The usage of ART was significantly associated with lower LBR regardless of the presence of a subfertility factor (p=0.012). Personalization of medical care using natural cycle or simple hormonal stimulation is associated with a significantly higher LBR than personalization including ART treatments regardless of maternal age and AMH level (OR= 2.9 CI 95% 1.03-8.88).

**Conclusion:**

Our study suggests that some endometrial immune profiles with targeted management of RPL are associated with a higher rate of LBR. ART may be negatively associated with LBR.

## Introduction

Recurrent pregnancy loss (RPL) is one of the most frustrating and difficult areas in reproductive medicine, since the etiology is often unknown and evidence-based diagnosis and treatment strategies are scarce. RRL is defined as three consecutive miscarriages before 20 weeks of gestation. Epidemiological studies have revealed that 1– 2% of women experience RPL (1, 2). In 2018, the ESHRE guideline reduced to two the number of spontaneous miscarriages required before initiating explorations.

Documented causes of this multifactorial, heterogeneous disorder include oocyte and sperm parameters, parental chromosomal structure, anatomical structure, immunological and thrombophilia factors (antiphospholipid syndrome, factor V Leiden, factor II homozygous mutation carriers), hormonal conditions and lifestyle, which implies that a multidisciplinary approach is required for the management of RPL (3–15).

Unexplained RPL is defined in the absence of a known cause and represents 50% of all RPL (16).

It is believed that a significant, although not exactly quantified, proportion of RPL is associated with immune etiologies (17) (18) and that in these cases the miscarriages can occur through persistent disturbances in several immune pathways (19). In this context, a major role could be played by the endometrium (20). Emerging evidences suggest that endometrial environmental dysregulations are related to some important reproductive failures among others Repeated Implantation Failure (RIF) and RPL (21–24). The endometrial immune profiling is a novel concept based on the analysis of the local immune reaction occurring in the endometrium at the time of the embryo implantation (25) (26) (27). The period of uterine receptivity called the implantation window occurs five to nine days after ovulation (28). During this specific period, a fundamental immune switch should occur locally to not only avoid the rejection of the semi allogenic embryo but also to promote its growth and nutrition (29) (30). The timed changes in the endometrial immune environment are essential for an adequate embryo implantation and placentation (31).

Our main hypothesis is that some miscarriages could be the consequence of early uterine immune dysregulations (21–24), which once documented could be corrected with personalized therapies. Uterine immune profiling through a better understanding of the endometrial environment seeks an optimum local balance for a successful implantation and gestation. Understanding the local uterine immune environment aims to anticipate the future interplay between the endometrium and the embryo. An underactive immune environment may fail to create the pseudo-inflammatory reaction required for a successful implantation. Conversely, an overactive immune environment may lead to the rejection of the embryo. Endometrial immune profiling has been initially designed to help infertile patients with history of RIF (27). By analogy, we here describe the clinical results observed in patients spontaneously fertile but with RPL.

The main objective of the study was to analyze the association of the endometrial immune dysregulations with LBR in women with a history of RPL. The secondary objective was to highlight the main prognostic factors for live births.

## Materials and Methods

### Protocol Approval and Patient Consent

In 2011, the Institutional Review Board and the Ethical Committee of St. Louis Hospital approved the prospective follow-up of a cohort after immune profiling in order to document their outcome and a potential benefit (ref. 2011- A00994-37).

Patients undergoing an endometrial biopsy provided their written informed consent allowing a uterine immune analysis and a prospective follow-up. All patients included in the ART program gave their informed consent before any fertility treatment (IVF/ICSI/Frozen Embryo transfer). We used this database to meet our main objective.

### Patients and Standard Screenings

This was an observational retrospective analysis of 104 patients with RPL, defined as at least three consecutives miscarriages following spontaneous pregnancies, who underwent an endometrial biopsy between January 2012 and December 2019. Patients initially had a standard RPL screening. If an anomaly was identified, it was corrected before an endometrial biopsy was performed after a recurrence of a new miscarriage.

Inclusion criteria were at least three miscarriages after spontaneous pregnancies, a complete screening including a three-dimension ultrasound scan, a uterine immune profiling and a follow-up over at least 6 months with personalized care if indicated after the assessment.

The exclusion criteria were as follows: patient refusing the therapeutic strategy following endometrial sampling, or patient with an incomplete screening. The collected data included: maternal age, BMI (Body Mass Index), smoking during pregnancy, previous number of miscarriages, the presence of endometriosis, adenomyosis (diagnosed by imaging, ultrasound or MRI) or tubal pathology, the ovarian reserve parameters (FSH (IU/L), LH (IU/L), Estradiol (pg/ml), AFC (Antral Follicular Count), AMH rate (ng/ml)), and the assessment of the uterine cavity. On the other hand, we collected information about their male partner and more specifically about their sperm quality. A low ovarian reserve was defined in this study as an AMH level below 1ng/mL and a maternal age over 41 years old.

Patients underwent the following standard check-up: screening for hereditary thrombophilia (Factor V and II mutations, Protein S assay, Methylene Tetra Hydro Folate Reductase (MTHRF) mutation, antiphospholipid, anticardiolipin and beta-2 glycoprotein I antibodies, parental karyotype, thyroid screening, ovarian reserve, hysteroscopy or hysterosonography, sperm evaluation with DNA fragmentation rate.

After confirming the endometrial anatomical integrity, we specifically assessed the endometrium with a 3-D pelvic scan combined to an endometrial biopsy during the mid-luteal phase. The 3-D pelvic scan detailed the endometrial proliferation and the uterine vascularization. We considered as a thin endometrium, a thickness below 7 mm or a volume below 2 ml.

### Endometrial Biopsy: Collection and Analysis

Endometrial biopsies were performed during the mid-luteal phase on a substituted cycle after 5 to 9 days of progesterone intake, or on a monitored cycle. The endometrial samples were gently aspirated by rotating a Pipelle de Cornier within the endometrial cavity. The Pipelle content was divided into two parts. The first part was placed in 4% formaldehyde (QPath Formol 4% buffered, VWR Chemicals, Fontenay-sous-Bois, France) for endometrial histological datation and CD56 immunolabeling, and the second part was placed in RNA- Later stabilization solution for further immunological analysis by RT-qPCR. The samples were sent at room temperature by postal services.

After confirmation of the mid-luteal phase by histological analysis, the RNA was extracted and reverse-transcribed by RT-PCR. uNK cells mobilization was initially evaluated using immunochemistry labeling positive CD56 cells and is now evaluated by real time PCR. We quantified by quantitative real-time PCR seven targeted biomarkers (IL-18, IL-15, TWEAK, Fn-14, CD56 and the references genes) with the Light Cycler 480 SYBR Green I Master mix (Roche). IL-15/Fn-14 and IL-18/TWEAK mRNA ratios were used to document the immune endometrial environment in which the embryo will be transferred. A patent untitled “method for increasing implantation success in assisted fertilization” described the present invention as a method for determining a uterine receptivity profile in order to increase implantation success in assisted fertilization ((PCT/EP2013/065355). In the patent, we defined the norms of expression for our biomarkers in a fertile cohort and documented that an immune profile was reproducible from one cycle to another over a period of six months if no surgery or pregnancy occurred in the meantime.

### Determination of Endometrial Immune Profile

The endometrial immune profile was defined according to the local balance of the mRNA expression of IL-18/TWEAK, IL-15/Fn-14 and CD56 immunostaining or mRNA expression (25, 26). Thus, four types of profiles were defined:

1. A balanced/Normal immune activation profile characterized by IL-18/TWEAK and IL-15/Fn-14 mRNA ratios and CD56+ cell count in the same range that previously defined in the fertile cohort.
2. A low immune activation profile characterized by low mRNA ratios for IL-15/Fn-14 (reflecting immature uNK cells) or IL-18/TWEAK or an absence of uNK mobilization.
3. A high immune activation profile is characterized by high mRNA ratios of IL-18/TWEAK or IL-15/Fn-14, or a high CD56+ cell count.
4. A mixed profile is characterized by a high mRNA ratio of IL-18/TWEAK (excess of Th-1 cytokines) simultaneously with low IL-15/Fn-14 ratio (reflecting immature NK).

### Therapeutic Interventions

The therapeutic interventions were as follows:

-All the patients with a BMI over 30 benefitted from a dietary consultation and a follow-up
-All the patients with a TSH over 2.5 IU/ml were substituted with Levothyroxine
-Patients with inherited thrombophilia and high homocysteinemia benefitted from a vitamin B9 supplementation
-If the diagnostic hysteroscopy revealed a synechia, a polyp, a myoma or a malformation, then an operative surgery was performed
-If autoimmune antibodies were detected, the patient was referred to an internal medicine team for extended investigation and establishment of a treatment plan
-If the sperm fragmentation was over 20%, the patient was substituted with antioxidant and controlled
-If the endometrial thickness was below 7 mm or endometrial volume below 2 ml, a long-term treatment with Tocopherol and Pentoxifylline was established with a follow-up over 6 months
After correction of the above parameters, the endometrial biopsy was then performed:
-Regarding the endometrial immune profile, suggestions were organized in six sections as follows (**Table 1**):

**Table 1:** Summary of suggested therapeutics depending on the endometrial profile.

Suggestion of personalization/immune profile	No dysregulation	Low profile	High profile	Mixed profile
**Endometrial scratching**	No	Yes	No	Yes
**Immunotherapy**	No	No	Yes (therapeutic test)	Yes (therapeutic test)
**Luteal adaptation**	No	No	Yes	Yes
**Luteal hCG supplementation**	No	Yes	No	Yes
**Exposure** **to seminal plasma**	No impact	Yes	No	No

- Endometrial scratching in the mid-luteal phase of the cycle preceding the treatment cycle

Endometrial scratching was recommended in case of low IL-15/Fn-14 mRNA ratio interpreted as an immaturity of uNK cells. Endometrial scratching could enhance uNK cell maturation, which strongly depends on the adequate expression of IL-15 (32). Any type of local injuries (such as endometrial biopsy) performed during the mid-luteal phase of the cycle stimulates, during the following cycle, the subsequent expression of adhesion molecules and IL-15, *via* toll-like receptor pathways (33). Immaturity of uNK cells may contribute to RPL by the induced poor angiogenesis required for an adequate placentation.

b. Immunotherapy

Adjunction of immunotherapy was proposed in overactive and mixed profiles either to decrease Th-1 cytokines or to control the recruitment or over-activation of uNK cells into killer cells through IL-15. In this RPL context, the objective is to avoid the rejection of the pregnancy.

As the first-line treatment, glucocorticoids (GC) supplementation was recommended (34). Patients received 20 mg of prednisolone and vitamin E (an antioxidant, 1 g daily) from day 3 of ovarian stimulation until the pregnancy test. If pregnancy occurred, the GC were continued at full dose until 8 weeks after embryo transfer, then decreased gradually and stopped at 10 weeks.

In routine practice, we still lack precise indications for its use based on objective testing (35) (36, 37). Consequently, only a normalization of the immune profile under GC may attest of its beneficial effect.

The rationale to use GC in such immune profiles is based on previous reports linking them to:

-decreased levels of Th-1 cytokines, NK cytotoxicity, and hyperactivation in lymphokine-activated killer cells (38).
-limit the consequence of IL-15 mRNA overexpression (39).
-modulate the Th1/Th2 balance when it is predominated by Th1 cytokines (40).

Our team observed that GC increased the expression of immunoregulators such as TWEAK and Fn-14, which have been shown to prevent the cytotoxicity of uNK (41)

In case of resistance to GC, low molecular weight heparin (LMWH) was an alternative option for, given their previously documented anti-complement effect (42, 43). LMWH was initiated after ovulation with the introduction of the progesterone at iso dose. If pregnancy occurred, LMWH was continued until 10 weeks and stopped.

As a second line of treatment, we also evaluated the efficiency of intravenous slow perfusion of Intralipids^®^. Previous authors reported its interest to control the hyperactivation of circulating NK cells and to regulate a Th-1-predominant cytokine balance (44) (45). Our team observed and reported a reduction of endometrial Th-1 cytokines as well as a better control of uNK cells mobilization after this perfusion (41).

This perfusion (a single slow perfusion of 4% diluted Intralipid^®^, Fresenius-Kabi) was systematically performed under medical supervision in hospital on days 8-10 of the embryo transfer cycle. If pregnancy occurred, another slow perfusion was administered at 3 weeks after the embryo transfer and again after 7 weeks.

At this stage regarding the type of immunotherapy, only the normalization of the uterine profile under therapy could indicate the efficiency of any drug since we are unable to predict the response to therapy.

c. Luteal support alteration

Hormonal adaptation of the luteal phase was recommended in case of over-active and mixed profiles. Progesterone, beside its endocrine role, is a crucial mediator of the endometrial immune tolerance required for a successful pregnancy.

Progesterone influences the maternal immune system through distinct pathways:

-Including the production of progesterone-induced blocking factor (PIBF), which inhibits NK cell activity (46) and leads to Th-2-dominant cytokine production by maternal lymphocytes (47).
-The induction of galectin-1, a progesterone-induced molecule, essential for tolerogenic dendritic cells, which in return promote the expansion of IL-10-secreting regulatory T cells (48).

In case of detection of an over-active profile, we hence recommended a high luteal support with 1200 mg daily of vaginal progesterone intake, for its immunosuppressive properties. When the IL-18 expression was elevated, we also recommended an oral estradiol supplementation of 4 mg daily to downregulate its local expression as previously described after ovulation (49). The higher luteal support began after the ovulation (natural cycle) or on the day of oocyte retrieval in case of IVF. This was continued until 8 weeks after embryo transfer for pregnant women.

d. Chorionic gonadotrophin (hCG) supplementation during luteal phase

Previous authors demonstrated that hCG triggers both the proliferation and the maturation of uNK cells (50) and promotes local angiogenesis (51, 52). Physiologically produced by the embryo, hCG is directly involved in the local reaction through the induction of an adequate angiogenesis while controlling the activation of uNK cells at the maternal-fetal interface (53, 54). We recommended hCG supplementation during the luteal phase in case of low mobilization or immaturity of the uNK. A dose equivalent to 1500 IU was subcutaneously administered at 4, 6, and 8 days after the start of progesterone support, during the implantation window.

e. Sexual intercourse after the ET

By inducing the expression of pro-inflammatory cytokines and chemokines and the recruitment of immune cells, the seminal plasma may have a positive role in preparing the endometrium for the implantation (55–57). We therefore recommended sexual intercourse during the luteal phase in case of low-immune activation but did not recommend exposure to seminal plasma in over-activated and mixed profile.

### Outcomes Definition

Outcome was classified in three categories: live birth (defined as delivery of a viable infant at 22 weeks or more of gestation), early pregnancy loss (defined as a spontaneous fetal demise at less than 22 weeks of gestational age), and no pregnancy.

To evaluate the performance of the personalized care applied, we define as a success if the patients gave birth, as a failure if the patient either did not get pregnant or underwent a new pregnancy loss.

### Data Collection

All procedures performed in studies involving human participants were in accordance with the ethical standards of the institutional and national research committee, with the 1964 Helsinki Declaration and its later amendments or comparable ethical standards. Data were anonymously extracted from the Matricelab database including 1778 patients with successful endometrial immune analysis and a prospective follow-up after immune profiling. These data were extracted for extended analysis of the cohort of women followed in our reproductive medicine unit (Les Bluets Hospital - Paris) and diagnosed with RPL.

After the global screenings including uterine immune profiling, the therapeutic strategy was defined, and each patient was followed-up for 6 months.

We confirmed that the personalized therapeutic strategy was applied, documented the type of ART potentially used (monitored natural cycle, IUI, IVF, FET) and the outcome.

In case of IVF or a frozen embryo transfer, we collected the outcome of the first subsequent embryo transfer following the analysis. In case of IUI, we collected the outcome after 3 attempts. If IVF or IUI were not required, we collected the outcome within the next six months following the assessment with the suggested strategy.

### Statistical Analysis

Data analyses were performed using R studio 1.3.1056. Continuous variables are presented as mean ± standard deviation (SD) or median. Categorical variables were analyzed in Pearson’s chi-square test or chi-square with Yates correction, when appropriate. Continuous data were analyzed with the Student’s t-test (for normally distributed) and the Mann–Whitney U test (for non-normally distributed data). Results were considered statistically significant with p-value below 0.05. We carried out a first univariate analysis comparing the group not pregnant and obtaining a pregnancy with live birth on the maternal characteristics and the results of the initial complete check-up carried out. The second univariate analysis was carried out on the same variables but comparing three groups: no pregnancy, early pregnancy loss and live birth.

In order to evaluate the impact of the therapies on the achievement of pregnancy in these women, we carried out a univariate comparative analysis between the no pregnancy group and the live birth pregnancy group on these therapies (treatment related to MAP and treatment related to endometrial anomalies). We also carried out this analysis by adjusting for age and AMH, the main known factors influencing the success of a pregnancy.

## Results

### Cohort Characteristics

**Figure 1:**
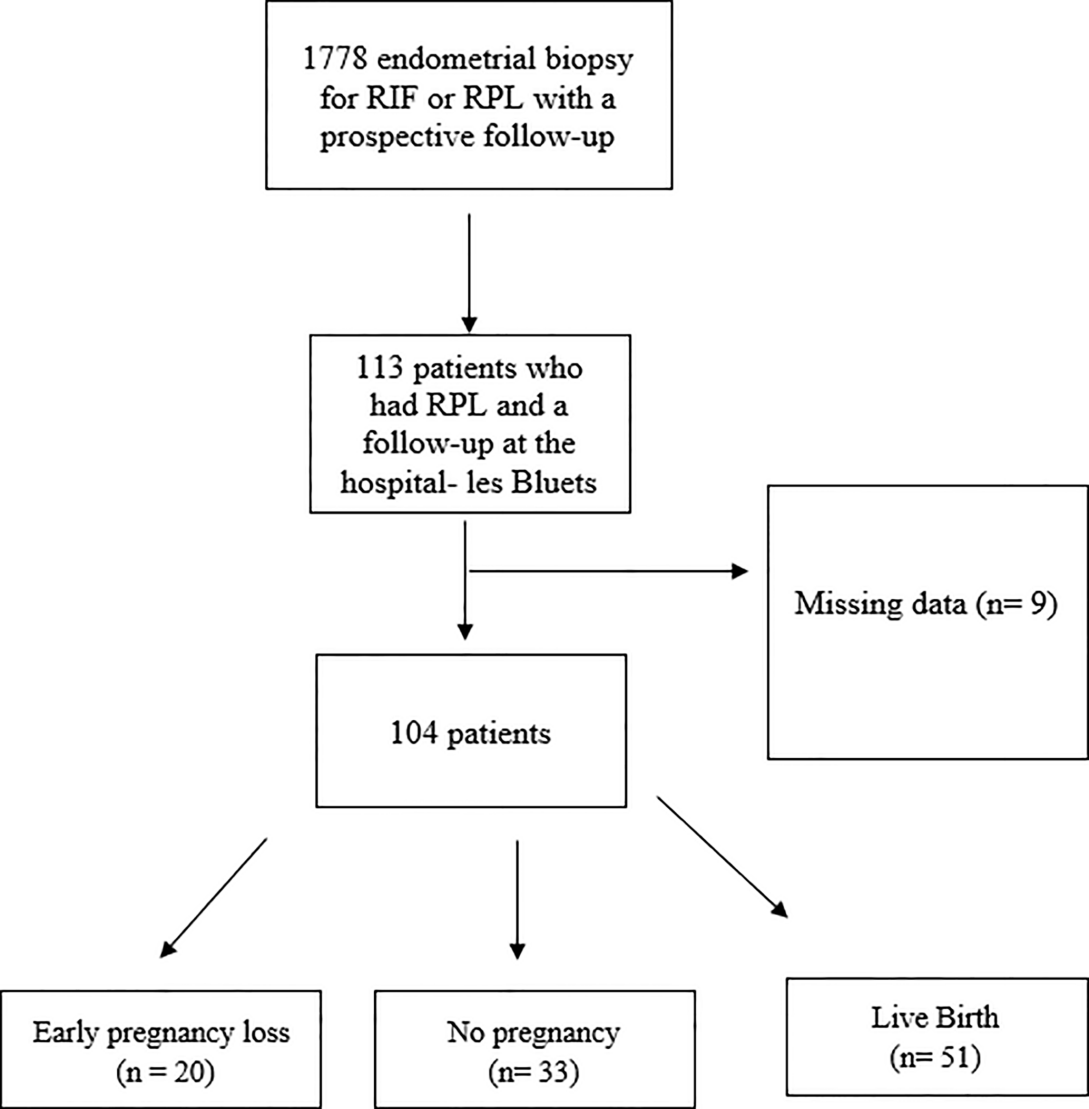
The process of our cohort selection.

Overall, between January 2012 and December 2019, 1778 patients underwent endometrial biopsy for RIF or RPL with prospective follow-up. 104 patients spontaneously fertile but who experienced RPL (at least 3 consecutives miscarriages) with a complete initial screening were included. The process o our cohort selection is detailed in **Figure 1**. Data was collected retrospectively.

The clinical and biological characteristics were compared in these three different groups (no pregnancy, recurrence of miscarriage, live birth) (**Table 2**). As expected, the three main factors significantly influencing the outcome were the maternal age (p<0.03), the number of previous miscarriages (p=0.008) and the presence of a low ovarian reserve (p=0.01). For women over 41 years old, the risk of a new miscarriage was 4 times higher than the chance of a live birth (73% vs 17%).

**Table 2:** Patients’ characteristics according to the outcome of pregnancy.

Characteristics^a^		Early pregnancy loss*(n = 20)	No pregnancy(n = 33)	Failure***(n = 53)	Live Birth**(n = 51)	p-value^b^	p-value^c^
**Age (years)**		**39 ± 3**	**37 ± 5**	**38 ± 4**	**38 ± 4**	0.007	**0.03**
	**< 35**	3 (9%)	8 (24%)	**11 (32%)**	**23 (68%)**	0.007	**0.03**
	**36-40**	10 (23%)	13 (30%)	**23 (52%)**	**21 (48%)**		
	**41 and more**	7 (27%)	12 (46%)	**19 (73%)**	**7 (17%)**		
**Smoking habits** **Current smoker**		3 (35%)	3 (25%)	**6 (50%)**	**6 (50%)**	0.94	**0.85**
**Ethnic group**						0.93	**0.95**
	**Europe**	10 (22%)	17 (37%)	**27 (59%)**	**19 (41%)**		
	**Others**	2 (13%)	7 (47%)	**9 (60%)**	**6 (40%)**		
**BMI (kg/m^2^)**		23 ± 4	25 ± 4	**25 ± 4**	**24 ± 3.5**	0.93	**0.98**
**BMI > 30 Kg/m^2^**		2 (20%)	3 (30%)	**5 (50%)**	**5 (50%)**	0.92	**1**
**Gravidity**		5 ± 3	5 ± 2	**5 ± 2**	**4 ± 1**	0.06	**0.12**
	**G3**	4 (15%)	5 (19%)	**9 (34%)**	**17 (66%)**	0.09	**0.27**
	**G4-G5**	12 (19%)	21 (34%)	**33 (53%)**	**29 (47%)**		
	**>/= G6**	4 (25%)	7 (44%)	**11 (69%)**	**5 (31%)**		
**Parity**		0.4 ± 0.6	0.6 ± 0.7	**0.5 ± 0.5**	**0.4 ± 0.7**	0.56	**0.56**
	**P0**	13 (20%)	18 (28%)	**31 (48%)**	**33 (52%)**	0.77	**0.90**
	**P1**	6 (18%)	12 (36%)	**18 (54%)**	**15 (46%)**		
	**>P2**	1 (14%)	3 (43%)	**4 (57%)**	**3 (43%)**		
**Duration of infertility (years)**		3.5 ± 4.2	4 ± 3	**4 ± 3.6**	**4 ± 5.2**	0.57	**0.58**
**Number of previous** pregnancy losses		5 ± 3.5	4 ± 1.5	**4 ± 2.4**	**3.6 ± 1**	0.11	**0.008**
	**=3**	6 (12%)	17 (33%)	**23 (45%)**	**29 (55%)**	0.30	**0.25**
	**4-6**	12 (27%)	13 (29%)	**25 (55%)**	**20 (44%)**		
	**>6**	2 (28%)	3 (43%)	**5 (71%)**	**2 (29%)**		
**Menstrual cycle length (days)**						0.51	**0.71**
	**< 27**	2 (28%)	1 (14%)	**3** (42%)	4 (58%)		
	**27-35**	17 (20%)	29 (34%)	**46 (54%)**	**40 (46%)**		
	**35-50**	1 (9%)	3 (27%)	**4 (36%)**	**7 (64%)**		
**OVARIAN RESERVE PARAMETERS**							
Day 3 FSH (IU/L)		7 ± 3	7 ± 3	**7 ± 3**	**7 ± 2**	0.97	**0.74**
	<7	14 (27%)	16 (31%)	**30 (58%)**	**22 (42%)**	0.27	**0.03**
	7-12	3 (7%)	16 (36%)	**19 (43%)**	**25 (57%)**		
	>12	2 (50%)	1 (25%)	**3 (75%)**	**1 (25%)**		
Day 3 LH (IU/L)		4.5 ± 2	10 ± 14	**8 ± 11**	**11 ± 29**	0.48	**0.45**
Day 3 Estradiol (pg/ml)		34 ± 17	39 ± 29	**38 ± 25**	**46 ± 58**	0.45	**0.53**
AFC		15 ± 8	11± 6	**13 ± 7**	**13 ± 6**	0.89	**0.36**
AMH (ng/ml)							
	>1	16 (20%)	22 (27%)	**38 (47%)**	**44 (53%)**	0.18	**0.22**
	0,5-1	2 (14%)	8 (57%)	**10 (71%)**	**4 (29%)**		
	< 0,5	2 (25%)	3 (38%)	**5 (63%)**	**3 (37%)**		
**SUBFERTILITY**							
Presence of Tubal obstruction		2 (16%)	5 (38%)	**7 (54%)**	**6 (46%)**	0.82	**0.86**
Associated endometriosis		1 (14%)	2 (29%)	**3 (43%)**	**4 (57%)**	0.71	**1**
Associated adenomyosis		2 (25%)	3 (37%)	**5 (62%)**	**3 (38%)**	0.72	**0.70**
Premature ovarian failure		10 (29%)	14 (41%)	**24 (70%)**	**10 (30%)**	0.01	**0.01**
Sperm abnormality		1 (14%)	1 (14%)	**2 (28%)**	**5 (72%)**	0.46	**0.81**
**History of prior uterine surgery**		1 (8%)	6 (46%)	**7 (54%)**	**6 (46%)**	0.82	**0.42**

BMI, Body Mass Index; FSH, follicle stimulating hormone; LH, luteinizing hormone; AFC, antral follicle count; AMH, antimüllerian hormone.

a Continuous data are presented as mean ± standard deviation; categorical data are presented as number (percentage).

b p value between the 2 groups (Failure/Live Birth).

c p value between the 3 groups (early pregnancy loss/no pregnancy/Live birth).

*Early pregnancy loss was defined as a spontaneous fetal demise at less than 12 weeks of gestational age.

**The live birth was defined as delivery of a viable infant at 22 weeks or more of gestation.

***The failure Group included early pregnancy loss and patients who have not had a pregnancy.

Despite a spontaneous fertility, a total of 52 patients had at least one element of subfertility (50%) probably influenced by our recruitment in a reproductive medicine unit. 13 patients had a tubal obstruction, 15 had endometriosis or adenomyosis, 34 had a low ovarian reserve, and a sperm abnormality was found in 7 male partners (defined as an asthenozoospermia, teratozoospermia or teratoasthenozoospoermia). A low ovarian reserve with an AMH below 1 ng/ml and a maternal age over 41 years old were the main factors contributing to the subfertility.

### Results of Conventional Screening

The results of the initial checkup are presented in **Table 3**. Balanced translocation and pericentric inversion were found in 2 patients. Heterozygous mutation of the MTHFR gene and increased homocysteinemia were identified in 8 patients. We found in 9 patients the presence of autoantibodies. In contrast, all the exploration focusing on the uterus (hysteroscopy, 3-D ultrasound and uterine immune profiling), the sperm fragmentation and the exploration of thyroid function were highly profitable to define subsequent strategy. A TSH level above 2.5 mIU/l was found in 89 patients (85.5% of the cohort). DNA fragmentation was pathological in 32% of the cases. 30% of patients required an operative hysteroscopy for trophoblast retention, synechia, polyp or fibroid. 31% had a thin endometrium in the mid-luteal phase.

**Table 3:** Prognostic factors of the outcomes pregnancy related to the global check-up.

Characteristics^a^	Early pregnancy loss* (n = 20)	No pregnancy (n= 33)	Failure*** (n=53)	Live Birth** (n= 51)	p-value^b^	p-value^c^
**Minor karyotype abnormalities**	0	1 (50%)	**1 (50%)**	**1 (50%)**	0.74	**0.77**
**Increased Homocysteine**	2 (25%)	2 (25%)	**4 (50%)**	**4 (50%)**	0.86	**0.55**
**Presence of autoimmune antibodies**	4 (44%)	0 (0%)	**4 (44%)**	**5 (66%)**	0.83	**0.08**
**TSH greater than 2,5**	18 (20%)	28 (31%)	**46 (51%)**	**43 (49%)**	0.70	**1**
**HYSTEROSCOPY**						
**Necessity of a surgery procedure**	7 (23%)	9 (30%)	**16 (53%)**	**14 (47%)**	0.93	**0.95**
**Malformation**	0 (0%)	0 (0%)	**0 (0%)**	**3 (100%)**	0.28	**0.63**
**Uterine ultrasound check-up** **Endometrial thickness (mm)**						
					0.73	**0.80**
**<7 mm**	7 (22%)	9 (28%)	**16 (50%)**	**16 (50%)**		
**>7mm**	13 (19%)	24 (35%)	**37 (54%)**	**32 (46%)**		
**Endometrial volume**					0.58	**0.86**
**< 2ml**	5 (18%)	9 (32%)	**14 (50%)**	**14 (50%)**		
**>2ml**	13 (20%)	23 (36%)	**36 (56%)**	**28 (44%)**		
**Sub endometrial VFI**	1.2 ± 1.6	1.2 ± 2	**1.2 ± 1.7**	**1.9 ± 4.1**	0.33	**0.57**
**Pulsatility Index (R +L)**	5.3 ± 4.4	4 ± 5	**4. 7± 4.2**	**5.5 ± 4.6**	0.31	**0.49**
**Diagnosis of Uterine Immune Profile**						
**Normal**	11 (42%)	7 (27%)	**18 (69%)**	**8 (31%)**	0.06	**0.02**
**Dysregulated**	9 (5%)	26 (33%)	**35 (45%)**	**43 (55%)**	0.01	**0.01**
**Low immune activation profile**	1 (4%)	11 (46%)	**12 (50%)**	**12 (50%)**		
**High/over immune activation profile**	7 (18%)	7 (18%)	**14 (36%)**	**25 (64%)**		
**Mixted profile**	1 (7%)	8 (53%)	**9 (60%)**	**6 (40%)**		
**Pathological sperm fragmentation index (>20%)**	9 (27%)	8 (24%)	**17 (51%)**	**16 (49%)**	0.36	**0.32**

a Continuous data are presented as mean ± standard deviation; categorical data are presented as number (percentage).

k Pearson’s Chi-square test.

^u^ Mann-Whitney test.

*Early pregnancy loss was defined as a spontaneous fetal demise at less than 12 weeks of gestational age.

**The live birth was defined as delivery of a viable infant at 22 weeks or more of gestation.

***The failure Group included early pregnancy loss and patients who have not had a pregnancy.

b p value between the 2 groups (Failure/Live Birth).

c p value between the 3 groups (early pregnancy loss/no pregnancy/Live birth).

### Outcome of Endometrial Immune Assessment

In this cohort, 75% of the patients suffered from an endometrial immune dysregulation. Within the patients with endometrial immune imbalance, 31% had a low uterine immune profile, 50% had an opposite pattern with a local immune over-activation and 19% had a mixed pattern.

In order to evaluate if the established strategies defined following our global screening was a success or a failure, we divided them in two main groups: those who gave a live birth (success group n=51) and those who failed to get pregnant or suffered a new miscarriage (failure group n=53) (**Tables 3** and **4**).

**Table 4:** Prognostic factors of the outcome’s pregnancy related to the endometrial treatments.

Characteristics ^a^	Failure***(n=53)	Live Birth** (n= 51)	OR	OR adjusted ^#^
**Treatment for endometrial thickness** **<7 mm and/or endometrial volume < 2ml**	**19 (51%)**	**18 (69%)**	0.98 [0.44-2.17]	1.10 [0.47-2.61]
**Diagnosis of Uterine Immune Profile**				
**Normal**	**18 (69%)**	**8 (31%)**		
**Dysregulated**	**35 (45%)**	**43 (55%)**	2.77 [1.10-7.44]	3.39 [1.27-9.84]
**Scratching/endometrial biopsy/hcg**	**12 (50%)**	**12 (50%)**	2.25 [0.72-7.38]	2.55 [0.75-9.24]
**Immunotherapy**	**14 (36%)**	**25 (64%)**	4.02 [1.43-12.1]	4.99 [1.65-16.5]
**Endometrial biopsy/hcg/immunotherapy**	**9 (60%)**	**6 (40%)**	1.5 [0.39-5.72]	2.07 [0.48-9.19]
**Treatment by immunotherapy (hight immune activation and mixted profile)**				
**Without therapy control**	**20 (47%)**	**22 (53%)**	2.73 [0,70-13,6]	2.38 [0.51-14.1]
**With therapy control**	**3 (25%)**	**9 (75%)**	1	1

a Continuous data are presented as mean ± standard deviation; categorical data are presented as number (percentage).

**The live birth was defined as delivery of a viable infant at 22 weeks or more of gestation.

***The failure Group included early pregnancy loss and patients who have not had a pregnancy.

^#^adjusted for age and AMH. Results from the univariate and multivariate analysis.

Uterine immune profiling was associated with a higher LBR if a dysregulation was identified and treated accordingly (55% vs 45%, p=0.01) (**Table 3**). On the contrary, a normal immune profile (resulting in an apparently balanced immune environment) was associated with a higher risk of a new miscarriage.

The **Table 3** shows the pregnancy outcomes related to the endometrial profile with adjusted results according to maternal age and AMH level. Compared with patients who had a normal profile, those with a dysregulated immune profile had an increased rate of live birth (OR 3.4 CI 95% 1.27-9.84) (OR = 5 CI 95% 1.65-16.5).

### Outcome When Using ART

ART was used in 86.5% (45 patients out of 52) of couples with at least one subfertility factor and in 32% of patients without identified subfertility factors. 7 patients with at least one subfertility factor had a spontaneous pregnancy. The usage of ART was significantly associated with lower LBR regardless of the presence of a subfertility factor (p=0.012). In fact, personalization of the medical care using their natural cycle or simple hormonal stimulation is associated with a significantly higher LBR than personalization including ART treatments regardless of maternal age and AMH level (OR= 2.9 CI 95% 1.03-8.88). If an IVF was performed, the prognosis factor was as expected the number of matures oocytes collected. A collection below 4 oocytes was associated with lower chance of LBR (p=0.03). These results are presented in **Table 5**.

**Table 5:** Prognostics factors related to the ART treatment characteristics.

Characteristics ^a^	Failure***(n=53)	Live Birth**(n= 51)	P-value	OR	OR adjusted ^#^
**Total number of art cycles**	1.4 ± 0.8	1.4 ± 0.8	0.89	1.02 [0.57-1.78]	0.95 [0.48-1.78]
**Stimulation protocols**					
**IVF- ICSI- FET**	33 (54%)	28 (46%)	0.012	1	1
**IUI**	13 (72%)	5 (28%)		0.51 [0.16-1.45]	0.62 [0.17-2.13]
**Spontaneous pregnancy- SOS**	7 (28%)	18 (72%)		3.05 [1.14-8.86]	2.89 [1.03-8.88]
**IVF protocol**					
**Antagonist**	16 (47%)	18 (53%)	0.69	1	1
**Long antagonist**	4 (57%)	3 (43%)		0.67 [0.12-3.47]	0.77 [0.12-4.81]
**No. of retrieved oocytes**	8.6 ± 5.2	8.3 ± 4.6	0.84	0.99 [0.86-1.13]	0.96 [0.81-1.12]
No. of (successful) oocytes***	3.6 ± 5	5.7 ± 5	0.10	1.08 [0.99-1.19]	1.10 [0.99-1.23]
**< 4**	27 (73%)	10 (27%)	0.03	1	1
**> 4**	16 (48%)	17 (52%)		2.87 [1.08-8.00]	3.64 [1.17-12.56]
Number of embryos transferred*	1.6 ± 0.5	1.6 ± 0.5	0.95	0.90 [0.38-2.12]	1.02 [0.36-3]
Age of embryo transferred*					
**D2-D3**	12 (54%)	10 (46%)	0.56	1	1
**D5-D6**	15 (53%)	13 (47%)		1.85 [0.48-7.48]	1.28 [0.23-6.97]
**D3 and D5**	0 (0%)	2 (100%)		1	1

ART, assisted reproductive technology; IVF, in vitro fertilization; ICSI, intracytoplasmic sperm injection; IUI, intrauterine insemination; SOS, simple ovarian stimulation; FET, frozen embryo transfer; NO, number.

a Continuous data are presented as mean ± standard deviation; categorical data are presented as number (percentage).

*For IVF-ICSI-FET.

**The live birth was defined as delivery of a viable infant at 22 weeks or more of gestation.

***The failure Group included early pregnancy loss and patients who have not had a pregnancy.

^#^adjusted for age and AMH.

Results from the univariate and multivariate analysis.

## Discussion

### Main Findings

The contribution of the endometrium appeared to be under-estimated in the management of RPL. The endometrial exploration in the mid luteal phase was not referred to in the ESHRE guidelines (58). In our study, 75% of RPL patients had a dysregulated immunological endometrial profile. After global screening, endometrial immune profiling was the only exploration to be significantly associated with a higher LBR if a dysregulation was identified and treated accordingly. Independently of age and AMH level, dysregulated immune profile is significatively associated with three times more LBR than normal profiles (OR=3.4 CI 95%1.27-9.84) or five times if overactivated and treated by immunotherapy after a test of sensitivity (OR = 5 CI 95% 1.65-16.5).

### Interpretation

#### Assessment of the Uterine Cavity Integrity and Endometrial Ultrasonic Development

Prior the evaluation in the mid-luteal phase, 29% of the cohort required a surgical procedure through an operative hysteroscopy. After control of the uterine cavity integrity, 35.6% had a thin endometrium at the 3-D scan performed in the mid-luteal phase and have been treated for it. As previously described, the evaluation of the endometrial volume in the mid luteal phase is more sensitive than the 2-D endometrial thickness to detect endometrial trophic problems (59–61). Thin endometrium (less than 7mm) is correlated with a decreased pregnancy rate and a higher miscarriage rate (60, 62–64). Using 3-D scan, thresholds of 2mL (65, 66) and 2.5mL (61, 67) define a poor endometrial development associated with a lower pregnancy rate. We did not find any significant difference regarding the outcome in patients with previous uterine surgery or thin endometrium, but they were all treated before the endometrial biopsy.

#### Endometrial Immune Assessment and Correction

Immune contribution of the endometrium appeared to be under-estimated in the management of RPL. Previous authors documented by flow cytometry or immunohistochemistry abnormal immune cell mobilization or expression in patients with either RIF or RPL suggesting that endometrial immune local dysregulations may contribute to implantation failures (68) (24) (69).

The endometrial immune profiling is based on RT-qPCR analysis of CD56 mRNA expression, IL-15/Fn-14 ratios (interleukin-15/fibroblast growth factor-inducible molecule) and IL-18/TWEAK ratios (interleukin-18/Tumor necrosis factor-like weak inducer of apoptosis), all factors known to intimately involved in the differentiation of the secretory endometrium to the receptive state.

By documenting the local immune response which must occur during the period of uterine receptivity, we seek to detect imbalances that can be modified to promote further embryo implantation. In our cohort, endometrial immune profiling was indeed the only exploration to be significantly associated with a significant higher LBR if a dysregulation was identified and treated accordingly. 75% of the patients had a dysregulated immunological profile and 55% of them have a live birth after the management of this dysregulation. 84.3% of live birth patients had a dysregulated and treated immune profile. Independently of age and AMH level, dysregulated immune profile is significatively associated with 3 times more live births than a normal profile (OR= 3.4 CI 95% 1.27-9.84) and more so, when overactivated and treated by immunotherapy, (OR = 5 CI 95% 1.65-16.5). Previous studies allegedly considered that some cases of RPL are related to immune etiologies (17). Extensive studies have been conducted to document the mechanisms of the endometrium receptivity to the embryo suggesting that the endometrium plays a major role in the RPL (20). Emerging evidences suggest that endometrial environmental dysregulations are related to some important reproductive failures among others RIF and RPL.

Our hypothesis is that re-balancing the initial dialog between the embryo and the endometrium may rescue some viable pregnancies. In patients with a local low activation, the stimulation of the maturation, mobilization, expression of uNK through uterine scratching, hCG luteal supplementation and exposition to seminal plasma aim to improve the mechanisms of angiogenesis and immunotropism (33, 34, 51, 52, 55, 57). In this case, the mechanism leading to the miscarriage would be a local deficiency of angiogenesis and immunotrophism. In patients with immune over-activation, the mechanism leading to the pregnancy loss would be the direct rejection of the embryo through an excessive activation of the immune cells and the introduction of immunotherapy including personalized luteal support aims to reverse the deleterious activity of immune cells to stop rejection (38–49).

A mixed profile is characterized by a high mRNA ratio of IL-18/TWEAK, the mechanism of rejection would be the combination of local hyper-activation leading to a defective angiogenesis for the placentation.

Clare Larsen and al (2) introduced the biosensor theory, where the endometrium has a role in embryo quality control, which may be less discerning in some women. Sporadic miscarriage can be seen as representing nature’s quality control system, preventing embryos with severe abnormalities in most cases from progressing beyond the peri-implantation period. Should this quality control be disrupted, such embryos may be allowed to establish implantation long enough to present as clinical pregnancy before failing, resulting in clinical miscarriage. The first study to demonstrate the biosensor function of decidualized endometrial stromal cells (ESC) showed that coculture with an arresting human embryo elicited a reduction in the production of key cytokine regulators of implantation including interleukin (IL)-1β, heparin-binding epidermal growth factor-like growth factor (HB-EGF), IL-6, and IL-10 (70). Women with RPL allow the implantation of low-quality embryos which will get rejected for pregnancy, in opposition to normally fertile women whose endometrium is more selective (71, 72). These theories lead to the concept of hyper-fertility of RPL patients with inability to select the embryos with live birth potential (59, 73, 74).

In other words, women who experience RPL may not be rejecting healthy embryos, but rather permitting embryos of low viability to implant long enough to present as a clinical pregnancy instead of being lost at a preclinical stage.

We may postulate that the re-balancing the endometrial environment, through the immune profiling method and personalized therapeutics, may affect positively the ability of the endometrial selection and could avoid the recrudescence of PRL with a major psychological impact for the couple.

#### Personalization With ART or Not?

The emotional impact of RPL and the urgency to conceive often lead couples to consider a variety of fertility treatments. In front of the impossibility to identify the cause behind RPL, treatments are often ART oriented.

As reported in our cohort, 33% of couples received ART without subfertility factor associated and ART has been an option in almost 80% of the cases.

However, personalization to optimize the uterine immune environment during spontaneous monitored cycles or with simple hormonal stimulation was significantly associated with a higher LBR than the sub-group who benefitted of personalization and ART, and this regardless of the maternal age and AMH level (OR= 2.9 CI 95% 1.03-8.88).

As age and ovarian reserve have a clear negative impact on RPL, ART may appear as a solution for patients but also for physicians to try to shorten the time to conceive. In 2019, a review conducted by Kirshenbaum and Orvieto documented that these empirical treatments do not improve the LBR in RPL patients (75). More specifically, for young fertile patients with RPL, Perfetto et al. (76) concluded that ART treatments had no positive impact and were not beneficial in order to reduce the time to pregnancy (71).

Maternal age appears crucial in the management of RPL with a clear negative impact on subsequent chance of LBR. Aneuploidy is probably one of the causes of RPL because it increases with women’s age and the rate of aneuploidy in blastocysts reaches 58% at the age of 40 years (77). But even PGT-A, a preimplantation genetic testing for aneuploidy, was not shown as strictly effective in preventing miscarriages in advanced maternal age (78). Optimizing the uterine environment may represent another strategy in these patients, facing failure despite using euploid embryos. A randomized control trial may also be interesting to conduct, evaluating the benefit or not of ART in spontaneously fertile patients with RPL associated to personalized care according to their endometrial immune profile.

## Conclusion

To conclude, our study suggests that endometrial immune profiling and management of RPL according to the endometrial dysregulations appear to be effective to increase subsequent LBR with simple and non-invasive therapeutic options. Such management appears to be effective independently of ART which is negatively associated with LBR. As maternal age and ovarian insufficiency are well-known major causes of RPL, we need to establish if ART is detrimental or not when this therapeutic personalization is applied according to endometrial immune profiling.

## Data Availability Statement

The original contributions presented in the study are included in the article/supplementary material. Further inquiries can be directed to the corresponding author.

## Ethics Statement

In 2011, the Institutional Review Board and the Ethical Committee of St. Louis Hospital approved the prospective follow-up of a cohort after immune profiling in order to document their outcome and a potential benefit (ref. 2011- A00994-37).

## Author Contributions

AK selected the RPL patients managed between 2012 and 2019, performed the immune profiling and wrote the manuscript with MC. MC collected the related clinical data, constructed tables and wrote the manuscript. LC performed with AK the immune analysis. LP-e and GD performed the endometrial biopsy and the 3-D US. AP and GK performed the statistical analysis. NL conceived the research with GK and MR, interpreted the uterine immune profiles to define personalized care. NL, GK and MR supervised the discussions and the writing of the manuscript. All authors contributed to the article and approved the submitted version.

## Funding

This study was supported by MatriceLab Innove for the diagnostic tests and the prospective collection of data for the follow-up.

## Conflict of Interest

NL created the MatriceLAB Innove SARL company and holds a patent covering the endometrial immune assessment test and appended recommendations (PCT/EP2013/065355). Authors AK and LC were also employed by MatriceLAB Innove SARL.

The remaining authors declare that the research was conducted in the absence of any commercial or financial relationships that could be construed as a potential conflict of interest.
